# A window-of-opportunity biomarker study of etodolac in resectable breast cancer

**DOI:** 10.1002/cam4.512

**Published:** 2015-08-15

**Authors:** Richard B Schwab, Shumei Kato, Brian Crain, Minya Pu, Karen Messer, Noel Weidner, Sarah L Blair, Anne M Wallace, Dennis A Carson, Barbara A Parker

**Affiliations:** 1 Department of Medicine, U.C. San Diego Moores Cancer Center3855 Health Sciences Dr., La Jolla, California, 92093; 2 Division of Biostatistics & Bioinformatics Department of Family & Preventative Medicine, U.C. San Diego Moores Cancer Center3855 Health Sciences Dr., La Jolla, California, 92093; 3 Department of Pathology, U.C. San Diego Moores Cancer Center3855 Health Sciences Dr., La Jolla, California, 92093; 4 Department of Surgery, U.C. San Diego Moores Cancer Center3855 Health Sciences Dr., La Jolla, California, 92093

**Keywords:** Biomarker, breast cancer, COX-2, cyclin D1, etodolac

## Abstract

Observational data show that nonsteroidal anti-inflammatory drug (NSAID) use is associated with a lower rate of breast cancer. We evaluated the effect of etodolac, an FDA-approved NSAID reported to inhibit cyclooxygenase (COX) enzymes and the retinoid X receptor alpha (RXR), on rationally identified potential biomarkers in breast cancer. Patients with resectable breast cancer planned for initial management with surgical resection were enrolled and took 400 mg of etodolac twice daily prior to surgery. Protein and gene expression levels for genes related to COX-2 and RXR*α* were evaluated in tumor samples from before and after etodolac exposure. Thirty subjects received etodolac and 17 subjects were assayed as contemporaneous or opportunistic controls. After etodolac exposure mean cyclin D1 protein levels, assayed by immunohistochemistry, decreased (*P* = 0.03). Notably, pre- versus post cyclin D1 gene expression change went from positive to negative with greater duration of etodolac exposure (*r* = −0.64, *P* = 0.01). Additionally, etodolac exposure was associated with a significant increase in COX-2 gene expression levels (fold change: 3.25 [95% CI: 1.9, 5.55]) and a trend toward increased *β*-catenin expression (fold change: 2.03 [95% CI: 0.93, 4.47]). In resectable breast cancer relatively brief exposure to the NSAID etodolac was associated with reduced cyclin D1 protein levels. Effect was also observed on cyclin D1 gene expression with decreasing levels with longer durations of drug exposure. Increased COX-2 gene expression was seen, possibly due to compensatory feedback. These data highlight the utility of even small clinical trials with access to biospecimens for pharmacodynamic studies.

## Introduction

Breast cancer remains the most common malignancy diagnosed among women in the world. Approximately 1.38 million women worldwide were diagnosed with breast cancer in 2008, accounting for 23% of all new cancers cases and 14% of cancer deaths 1. Breast cancer is now also the leading cause of cancer death among woman in economically developing countries. Thus, inexpensive, safe, and effective preventative and adjuvant therapies are urgently needed.

Nonsteroidal anti-inflammatory drugs (NSAIDs) are cyclooxygenase (COX)-1 and -2 inhibitors that are commonly used to treat pain, inflammation, and fever. In large epidemiological studies, NSAID use has been associated with a preventive effect in breast cancer 2,3. Overexpression of COX-2 in cancer is known to promote tumor growth via stabilization and nuclear translocation of *β*-catenin which then leads to expression of growth-promoting genes 4. Thus, the inhibition of COX-2 by NSAIDs is considered to be one of the main mechanisms that may lead to anticancer activity 5. Several studies have also demonstrated that various NSAIDs have off-target, COX-independent, anticancer activities which include inhibition of peroxisome proliferator-activated receptors (PPAR) and nuclear factor kappa-light-chain-enhancer of activated B cells (NFkB) pathways 6,7. Investigating safe and inexpensive therapeutic options for breast cancer treatment and prevention may benefit patients particularly where medical resources are constrained.

Despite the possible anticancer and preventive effects in breast cancer 8–11, studies with these agents have been limited in part by reported cardiovascular risks associated with their use 12,13. Considering that long-term therapy is required in the adjuvant setting or as a part of cancer prevention strategies, candidate prevention or treatment medications will need substantial clinical safety data to be considered for study. Etodolac, an FDA-approved NSAID, has excellent postmarketing safety data with gastrointestinal disturbances being the most frequently reported side effects 14–16.

In addition to inhibiting COX-2 17, etodolac has COX-independent activities including inhibiting retinoid X receptor (RXR*α*) leading to apoptosis in cancer cells with high expression levels of the PPAR*γ*/RXR*α* nuclear receptor complex 18. Etodolac negatively regulates PPAR*γ* function which then downregulates cyclin D1 leading to tumor growth inhibition 19. Notably, PPAR*γ* is known to serve as a tumor promoter in the mammary gland leading to tumor development 20. Overall, the potential antitumor effect and safety profile of etodolac make it a good candidate for study in the preventative or therapeutic setting.

To investigate the biomarker effects of etodolac in breast cancer, we conducted a window-of-opportunity study in patients with resectable breast cancer planned for initial management with surgery. Patients were given etodolac at standard doses prior to surgery, and tumor tissue samples obtained before and after the etodolac exposure were evaluated for COX-2, RXR*α*, and related gene expression. When technically feasible, protein expression was also assayed. The central aim of this study was to evaluate if etodolac exposure would alter rationally identified biomarkers in women with resectable breast cancer.

## Material and Methods

### Patients

Following study review and approval by the UCSD Human Research Protections Program, patients were screened at the time of presentation to our breast surgery clinic with either an abnormal clinical breast examination or an imaging study. Patients with history of bleeding disorder, gastrointestinal bleeding, NSAID-induced asthma, NSAID hypersensitivity, or current need for anticoagulation were excluded. Patients requiring antiplatelet agents other than ≤325 mg of aspirin per day were also excluded. Consented subjects who were found to have resectable breast cancer and planned for surgical resection at UC San Diego were given study drug. Four opportunistic controls were available from subjects that did not receive study medication. Under separate approval an additional 13 anonymous contemporaneous controls were identified by pathology based on date of surgery falling within the study time frame, UCSD samples from biopsy and final surgery, and review of medical records indicating no NSAID use at the time of cancer diagnosis or prior to surgical resection. Most prospectively consented subjects had flash frozen tumor samples collected at the time of biopsy and time of surgical resection; all other tumor samples used were standard of care formalin-fixed, paraffin-embedded, and only compatible with immunohistochemical assays.

#### Treatment and evaluation

Eligible patients were started on etodolac 400 mg orally twice daily as soon as eligibility was determined, typically shortly after pathologic confirmation of breast cancer. The study drug was continued until 2 days prior to surgery. Surgeries were not delayed to allow for a specific duration of drug exposure. Breast cancer tumor specimens before and after the intervention were evaluated for gene expression levels of the COX-2 pathway (COX-2 and *β*-catenin) and the RXR*α* pathway (RXR*α*, PPAR*γ*, and cyclin D1) as well as cyclin D1 protein level by immunohistochemistry. Preexposure samples were collected at the time of diagnostic biopsy and flash frozen in liquid nitrogen. Postexposure samples were collected by a licensed member of the UCSD pathology department during immediate gross examination of the resected tumor specimen and also flash frozen in liquid nitrogen. Samples were stored in liquid nitrogen until subsequent quantitative polymerase chain reaction (qPCR) assay.

### Tissue RNA extraction

Prior to RNA extraction a portion of each snap frozen tissue was fixed in formalin, paraffin-embedded, and H&E stained to determine the presence of tumor. The remaining tissue was transitioned into Ambion RNA-later-ICE (Carlsbad, CA) and stored at −20°C. RNA stabilized tissue was processed using Qiagen's RNeasy Lipid Tissue Mini Kit (Valencia, CA). RNA was subsequently DNase treated using Ambion Turbo DNA free (Carlsbad, CA) and evaluated using a Nanodrop spectrophotometer (Waltham MA). Measured amounts of RNA were carried forward for cDNA synthesis using Bio-Rad iScript (Hercules, CA) per manufacturer's recommendations.

### Quantitative PCR

Quantification of gene expression was performed using hydrolysis probes selected from the Roche Universal Probe Library (Basel, Switzerland). Primer sequences and probe combinations were determined using the Roche ProbeFinder version 2.10. Primers were purchased from Integrated DNA Technologies (San Diego, CA) with the following primer sequences and paired with the corresponding probe number: PPAR*γ* (NM_138712.2) gacctgaaacttcaagagtaccaaa and tgaggcttattgtagagctgagtc, probe #39; RXR*α* (NM_002957.3) acatgcagatggacaagacg and gagagccccttggagtcag, probe #26; CCND1 (NM_053056.2) gaagatcgtcgccacctg and gacctcctcctcgcacttct, probe #67; CTNNB1 (*β*-catenin) (NM_001904.2) tgttaaattcttggctattacgaca and ccaccactagccagtatgatga, probe #8; COX-2 (NM_000963.1) tgggaagccttctctaacctc and tcaggaagctgctttttacctt, probe #69; KRT7 (NM_005556.3) atcgagatcgccacctacc and actccatctccagccaacc, probe #67; and 18S (M10098.1) ctcaacacgggaaacctcac and cgctccaccaactaagaacg, probe #77. Reactions were plated in duplicate in a total volume of 25 *μ*L using concentrations of 200 and 100 nmol/L of the corresponding primers and probe, respectively. Reactions were conducted with ABI Taqman Master Mix (Carlsbad, CA) under Universal Cycling Conditions and data collected on the Bio-Rad iCyclerIQ (Hercules, CA). Fold changes were calculated using the ΔΔCt method. Experimental genes of interest were normalized to the reference gene, 18S, and subtracted by the presurgical ΔCt in order to calculate the fold change post exposure.

### Immunohistochemistry

Immunohistochemistry for cyclin D1 was performed by the UC San Diego Medical Center Immunohistochemistry laboratory using cyclin D1 rabbit monoclonal antibody CRM307C from Biocare Medical (Concord, CA). Prior to staining, slides underwent antigen retrieval with Biocare Medical Borg Decloaker high-pH buffer (Concord, CA) and heat-induced epitope retrieval in a Biocare Decloaking Chamber (Concord, CA). Primary antibody was diluted 1:60 with Biocare Renoir Red diluent (Concord, CA) and incubated for 35 min.

### Statistical analysis

To compare the two groups, a Wilcoxon rank sum test was used for a continuous variable and a Fisher's exact test was used for a categorical variable. A Wilcoxon signed rank test was used to assess if there were significant changes in values before and after treatment within a group. To calculate fold changes in gene expression pre- and posttreatment for these etodoloc-treated patients, geometric means and their 95% confidence intervals were provided. A Spearman rank correlation test was used to assess the correlation between gene expression changes and treatment duration.

## Results

### Baseline characteristics

A total of 47 patients with resectable breast cancer participated in this study. Thirty subjects were given etodolac and 17 patients were used as opportunistic/contemporaneous controls. The majority of patients in both groups had infiltrating ductal carcinoma, while a few subjects had ductal carcinoma in situ, mixed invasive ductal and lobular carcinoma, invasive lobular carcinoma, or malignant phyllodes tumor (Table1). Most patients had estrogen receptor (ER)/progesterone receptor (PR)-positive and Her2-negative receptor status (Table1).

**Table 1:** Patient Characteristics

	Etodolac group(*N* = 30)	Control group(*N* = 17)	*P*-value
Age (mean ± SD)	59 ± 12	62 ± 9	0.38
Histology, *N* (%)			0.33
IDCA	21 (70)	11 (65)	
DCIS	4 (13)	5 (29)	
Other^1^	5 (17)	1 (6)	
Receptor status, *N* (%)			0.77
Triple positive	1 (3)	0 (0)	
ER + /PR + /Her2−	15 (50)	12 (71)	
ER − /PR − /Her2+	4 (13)	2 (12)	
ER + /PR − /Her2−	5 (17)	1 (6)	
Triple negative	5 (17)	2 (12)	

IDCA is invasive ductal carcinoma, DCIS is ductal carcinoma in situ. ER is estrogen receptor and PR is progesterone receptor. *P*-value evaluated by Fisher's exact test.

1 Other: mixed invasive ductal and lobular carcinoma (*N* = 3), invasive lobular carcinoma (*N* = 2), phyllodes (*N* = 1).

### Safety

Subjects were assessed for adverse events according to the National Cancer Institute Common Terminology Criteria version 3 prior to surgery and, when relevant, after 4 weeks on study drug. One expected grade 3 adverse event, allergic reaction, occurred after two doses of etodolac. This subject was given intravenous steroids and antihistamines with rapid resolution of her symptoms. Her symptoms did not recur and the subject was not hospitalized. A second subject stopped study drug due to grade 1 stomach pain after 2 days on drug. No additional intervention was required beyond discontinuation of study drug. No excessive bleeding at time of surgery was noted for any subjects.

### Change in cyclin D1 protein level after etodolac exposure

**Figure 1:**
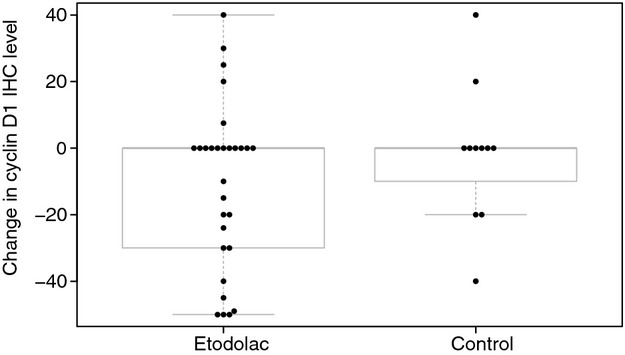
Dot plot for changes in cyclin D1 protein immunohistochemical level after treatment by cohort. There are 11 zero values in the etodolac cohort and six in controls. The immunohistochemical values were scored from 0 to 100.

Cyclin D1 protein level by immunohistochemistry was evaluated in surgical samples collected before and after etodolac exposure. We were able to obtain both pre- and postsurgical samples in 29 patients who were given etodolac and 11 patients without intervention. Median duration of etodolac treatment was 17 (range 1–39) days. Immunohistochemically stained slides were evaluated and scored from 0 to 100 (100 being strong positive) using a CompuCyte iCys laser scanning cytometer (Austin, TX). As expected, after etodolac exposure, cyclin D1 decreased significantly (*etodolac*: mean decrease 10.7, 95% CI [1.26, 20.16]; *control*: mean decrease 1.82, 95% CI [−12.2, 15.85]) (Fig.1), while the magnitude of the decrease did not differ significantly between the exposed and the control group, possibly due to the small number of controls. Blinded categorical pathology review of these same immunohistochemical slides correlated with the iCys analysis (Spearman rank correlation = 0.67, *P* < 0.00001, data not shown).

### Change in gene expression levels of COX-2 and RXR*α* pathways after etodolac exposure

In subjects exposed to etodolac with pre- and postexposure flash frozen tumor available (*n* = 15), we evaluated the gene expression levels associated with the COX-2 pathway (COX-2 and *β*-catenin) and the RXR*α* pathway (RXR*α*, PPAR*γ*, and cyclin D1). Each gene expression level from pre- and postetodolac treatment samples was normalized to housekeeping genes and evaluated for the fold change in gene expression level before and after the etodolac exposure. Ribosomal RNA 18S and cytokeratin-7 were used as control genes and the results were similar using either gene (data presented using 18S).

**Figure 2:**
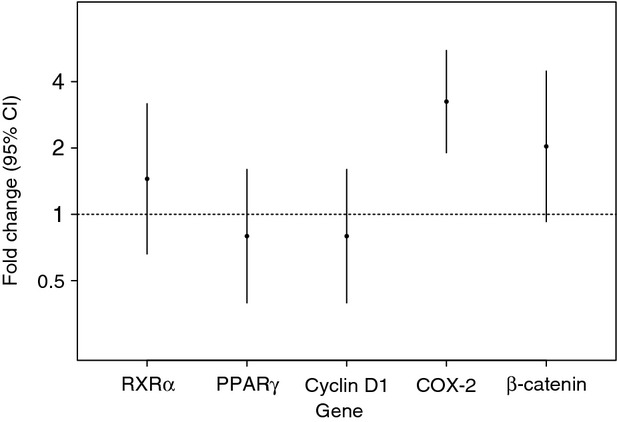
Fold change in gene expression levels associated with COX-2 pathway (COX-2 and *β*-catenin) and RXR*α* pathway (RXR*α*, PPAR*γ*, and cyclin D1) before and after etodolac treatment. *P < 0.001. COX-2, cyclooxygenase-2; RXR*α*, retinoid X receptor alpha; PPAR*γ*, peroxisome proliferator-activated receptor gamma.

No significant changes were observed in overall expression levels of RXR*α* pathway genes after etodolac exposure (Fig.2). Fold changes, RXR*α*: 1.45 [95% CI: 0.66, 3.18], *P* = 0.59; PPAR*γ*: 0.8 [95% CI: 0.4, 1.6], *P* = 0.47; or cyclin D1: 1.14 [95% CI: 0.44, 3.0], *P* = 0.95). However, we did observe effects in the COX-2 pathway with a significant increase in COX-2 gene expression level (3.25 [95% CI: 1.9, 5.55], *P* < 0.001), and a near significant increase in *β*-catenin (2.03 [95% CI: 0.93, 4.47], *P* = 0.07).

### Duration of etodolac exposure and change in gene expression levels of COX-2 and RXR*α* pathways

**Figure 3:**
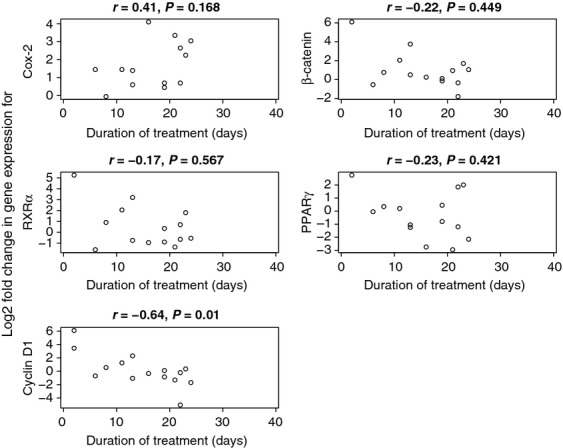
Correlation of COX-2 pathway (COX-2 and *β*-catenin) and RXR*α* pathway (RXR*α*, PPAR*γ* and cyclin D1) gene expression levels with the duration of etodolac exposure. COX-2, cyclooxygenase-2; RXR*α*, retinoid X receptor alpha; PPAR*γ*, peroxisome proliferator-activated receptors gamma.

Among etodolac-exposed subjects, we compared the duration of treatment with the change in gene expression level of COX-2 and RXR*α* pathway genes. We did not observe a significant correlation in the following genes; COX-2 (*r* = 0.41, *P* = 0.17), *β*-catenin (*r* = −0.22, *P* = 0.45), RXR*α* (*r* = −0.17, *P* = 0.57), or PPAR*γ* (*r* = −0.23, *P* = 0.42) (Fig.3). However, cyclin D1 demonstrated a statistically significant inverse correlation of gene expression change with duration of etodolac exposure (*r* = −0.64, *P* = 0.01) (Fig.3).

## Discussion

Assay of a limited set of rationally identified genes, evaluated before and after etodolac exposure, found that etodolac is associated with a decrease in cyclin D1 protein level as assayed by immunohistochemistry. We also found that cyclin D1 gene expression decreased with longer duration of etodolac exposure. These results are in agreement with previous preclinical study 19 and confirm the activity of etodolac on cyclin D1 levels in vivo in patients with breast cancer. Our data suggest that etodolac may have utility targeting cyclin D1 in breast cancer and that temporal effects should be considered in using tumor gene expression levels of cyclin D1 as a biomarker.

We also observed an increase in COX-2 pathway (COX-2 and possibly *β*-catenin) gene expression after etodolac exposure. Although etodolac is a well-known selective COX-2 enzymatic inhibitor 17, compensatory increased gene expression of COX-2 with NSAIDs has been reported in the past 21,22. Additional study, such as analysis of prostaglandin E2 levels, will be required to determine if the increased gene expression level of COX-2 after etodolac exposure is associated with preserved enzymatic activity.

Our results for COX-2 demonstrate the need for optimized biomarkers to monitor the effect of agents that may be subject to compensatory responses. The compensatory effect seen in this study may partially explain why previous studies evaluating NSAIDs in breast cancer prevention have had conflicting results 23,24. Limitations of our study include its small sample size and the lack of protein or functional assays of the COX2 pathway. A major strength of our approach is its potential clinical relevance. By conducting this biomarker study in patients with breast cancer, we have, by definition, controlled for the tumor microenvironment, pharmacokinetics of study drug, and other unknown factors which can generate misleading results in model systems 25. To the best of our knowledge, this is the first clinical observation of a COX-2 increase in gene expression with etodolac treatment. Notably, feedback gene expression upregulation has been reported with other inhibitors such as a BRAF inhibitor which causes upregulation of EGFR gene expression in colon cancer 26, in this case leading to therapeutic resistance.

Recent work has generated an abundance of targeted therapies for testing in early phase clinical trials. However, most of these drugs fail to demonstrate efficacy and relatively few go into phase III study. To increase the probability of success, pharmacodynamic results from preclinical work should be validated in patients before conducting larger clinical trials. The currently accruing I-SPY 2 trial 27 not only tests the efficacy of investigational drugs in the neoadjuvant setting for breast cancer but also includes confirmatory and discovery biomarker testing. Studies, such as the small study presented here and the large ongoing I-SPY 2 trial, address challenges to developing new preventative and targeted treatments for cancer.
